# Diosmin, a Citrus Nutrient, Activates Imidazoline Receptors to Alleviate Blood Glucose and Lipids in Type 1-Like Diabetic Rats

**DOI:** 10.3390/nu9070684

**Published:** 2017-06-30

**Authors:** Chia-Chen Hsu, Mang Hung Lin, Juei-Tang Cheng, Ming Chang Wu

**Affiliations:** 1 Department of Food Science, College of Agriculture, National Pingtung University of Science and Technology, Pingtung City 90801, Taiwan; r1221508@ms19.hinet.net (C.-C.H.); manghung2468@gmail.com (M.H.L.); 2 Chief Secretary’s Office, Chiayi Hospital, Ministry of Health and Welfare, Chiayi City 60001, Taiwan; 3 Department of Medical Research, Chi-Mei Medical Center, Yong Kang, Tainan City 73101, Taiwan; 4 Institute of Medical Science, College of Health Science, Chang Jung Christian University, Guei-Ren, Tainan City 71101, Taiwan

**Keywords:** diosmin, β-endorphin, imidazoline receptor, STZ-diabetic rats

## Abstract

Diosmin is a nutrient that is widely contained in citrus and that has been indicated to improve glucose metabolism in diabetic disorders. Recently, we demonstrated that diosmin induces β-endorphin to lower hyperglycemia in diabetic rats. However, the mechanisms of diosmin in opioid secretion were unclear. Therefore, we focused on the secretion of opioids from isolated adrenal glands induced by diosmin. The changes in the released β-endorphin-like immunoreactivity (BER) were determined using ELISA. Diosmin increased the BER level in a dose-dependent manner, and this effect was markedly reduced in the absence of calcium ions. Activation of the imidazoline I-2 receptor (I-2R) has been introduced to induce opioid secretion. Interestingly, we observed that diosmin activates CHO cells expressing I-R. Additionally, diosmin-increased BER was inhibited by the blockade of I-2R in isolated adrenal glands. Additionally, an antagonist of I-2R blocked diosmin-induced effects, including the reduction in hyperglycemia and the increase in plasma BER in streptozotocin-induced diabetic rats (STZ-diabetic rats). Repeated treatment of STZ-diabetic rats with diosmin for one week induced changes in hepatic glycogen, lipid levels, and the expression of phosphoenolpyruvate carboxykinase (PEPCK). Furthermore, an antagonist of I-2R blocked the diosmin-induced changes. Additionally, plasma lipids modified by diosmin were also reversed by the blockade of I-2R in STZ-diabetic rats. Taken together, we suggest that diosmin may activate I-2R to enhance the secretion of β-endorphin from adrenal glands and to influence metabolic homeostasis, resulting in alleviation of blood glucose and lipids in STZ-diabetic rats.

## 1. Introduction

Diabetes is a major public health problem throughout the world. The classic symptoms of diabetes include polydipsia, polyuria, polyuria, weight loss, fatigue, irritability, and blurred vision. Currently available therapies for diabetes include insulin and hypoglycemic agents. Therefore, the search for more effective and safer hypoglycemic agents is important. Recently, some natural products have been documented to be useful in the treatment of diabetes. Diosmin, a natural flavone glycoside (diosmetin 7-rutinoside; PubChem CID 5281613), is contained mainly in citrus [1] and it shows antihyperglycemic [2] and anticancer effects [3], in addition to anti-inflammatory and antioxidant-like actions [4]. In type-2 diabetic animals, diosmin attenuated hyperglycemia and increased insulin secretion [5]. A new complex, Zndiosmin, has also been developed to improve another model of type-2 diabetic rats [6]. Recently, we demonstrated that diosmin induces β-endorphin secretion from the adrenal gland to reduce hyperglycemia in streptozotocin-induced diabetic rats (STZ-diabetic rats) [7]. However, the mechanisms of action of diosmin remain unknown. 

As described previously [8], β-endorphin, which is involved in the lowering of hyperglycemia, was induced by herbal products including flavonoids in STZ-diabetic rats, an animal model of type-1 diabetes. The secretion of β-endorphin from the adrenal gland is mainly regulated by imidazoline receptors [9]. Therefore, we are interested in understanding the role of the imidazoline receptor (I-R) in the effects of diosmin. The present study focused on this and was designed to confirm our hypothesis that diosmin activates I-R to induce opioid secretion for the reduction of hyperglycemia in STZ-diabetic rats.

## 2. Materials and Methods

### 2.1. Materials

Diosmin (purity > 94%) and streptozotocin (purity > 98%) were purchased commercially from Sigma-Aldrich Chemical Co. (St. Louis, MO, USA). The commercial kit for β-endorphin-like immunoreactivity (BER) was purchased from Peninsula Laboratories (Belmont, CA, USA). The ECL-Western blotting system was purchased from Amersham Biosciences UK, Ltd. (Buckinghamshire, UK). The stock solution of diosmin was prepared in dimethylsulfoxide (DMSO). A fresh solution diluted with 9% normal saline to the indicated dose was then employed to treat the animals.

### 2.2. Animal Model

Male Sprague–Dawley (SD) rats weighing 250 to 280 g were obtained from the National Laboratory Animal Center (Taipei, Taiwan). STZ-diabetic rats were induced by intravenous injection (i.v.) of STZ (65 mg/kg), as described previously [10]. Animals were considered diabetic if they had a plasma glucose concentration of 310 mg/dL or greater, in addition to polyuria and other diabetic features. All studies were performed two weeks after the injection of STZ. All experiments were performed under anesthesia with sodium pentobarbital (35 mg/kg) via intraperitoneal injection (i.p.), and all efforts were made to minimize animal suffering. All experimental procedures performed in studies involving animals were approved by the Local Ethics Commission for Animal Experiments of Chi-Mei Medical Center (No. 105110331) and were in accordance with the 1996 NIH Guide for the Care and Use of Laboratory Animals. 

### 2.3. Experimental Design

Normal and STZ-diabetic rats were divided into five groups with eight rats in each. Group 1, normal rats received saline; Group 2, STZ rats received saline; Group 3, STZ rats received diosmin (160 mg/kg, i.p.); Group 4, STZ rats received a 30-pretreatment with BU224 (0.5 mg/kg, i.p.) and then administered with diosmin (160 mg/kg, i.p.), Group 5, STZ rats pretreated with BU224 (1 mg/kg, i.p.) 30 min before the administration of diosmin (160 mg/kg, i.p.). In continuous study, all treatments were performed once daily for 7 days, as described previously [7]. 

The food intake and bodyweight were measured daily. Blood glucose level was determined using the overnight fasted animals. Rats were anesthetized with sodium pentobarbital (35 mg/kg, i.p.), and blood samples were collected from the tail vein. Then, the rats were sacrificed by cervical dislocation, and the liver was isolated and stored at −80 °C for subsequent analysis.

### 2.4. Determination of Plasma Glucose, Insulin, Lipid, and BER Levels

The concentration of plasma glucose was measured by the glucose oxidase method using an analyzer (Quik-Lab, Ames, IA, USA; Miles Inc., Elkhart, IN, USA). The insulin level was examined using a Mercodia insulin ELISA kit (Mercodia AB, Uppsala, Sweden). Total lipids in liver were extracted according to the previous method [11]. The levels of lipids were determined using a Fuji Dri-Chem Slide TCHO for cholesterol and a Slide TG for triglyceride to read in a machine of Fuji Dri-Chem 400i (Fujifilm, Tokyo, Japan). In addition, the concentration of BER was estimated using a commercially available ELISA kit (Peninsula Laboratories, Belmont, CA, USA). 

### 2.5. Glycogen Extraction and Assay

For glycogen extraction, frozen liver tissue (10 mg) was homogenized in 200 µL of ice-cold water. Equal amount (5 μL) of the tissue lysates was used for assay of glycogen content using a Glycogen Assay Kit (Abcam, Cambridge, MA, USA). 

### 2.6. Isolation of Adrenal Gland 

The adrenal glands were obtained from STZ-diabetic rats after sacrificed, and the medullae were immediately dissected as described previously [9]. Tissues were placed in an incubator for a 15 min pre-incubation at 37 °C and bubbled with air (95% O_2_ and 5% CO_2_) under continuous agitation with 2 mL modified Krebs solution, as in our previous method [9]. Calcium-free Krebs solution was prepared in the same manner, except for the addition of calcium chloride, meaning that calcium ion was not included. Then, the tissues were transferred to fresh incubation tubes with or without an antagonist of imidazoline I-2 receptor (I-2R), incubated for 15 min at 37 °C, and further incubated with diosmin for another 30 min under continuous agitation (40 cycles/min) [9]. Placing the tubes on ice terminated the incubation. The incubated medium was then collected and frozen at −70 °C until the assay for β-endorphin-like immunoreactivity (BER).

### 2.7. Cell Cultures

CHO-K1 cells (BCRC No. CCL-61) purchased from the Culture Collection and Research Center of the Food Industry Institute (Hsin-Chiu City, Taiwan) were maintained in growth medium composed of F-12K supplemented with 10% fetal bovine serum. Cells were subcultured once every 3 days by trypsinization (GIBCO-BRL Life Technologies, Gaithersburg, MD, USA), and the medium was changed every 2–3 days.

### 2.8. Transfection of Imidazoline Receptor Gene in CHO-K1 Cells

Nischarin (NISCH) is a mouse homolog of human imidazoline receptor antisera-selective (IRAS) protein, which binds to the cytoplasmic domain of integrin α5/β1 [12] and may serve as a functional imidazoline receptor [13]. Following our previous method [13], CHO-K1 cells were transiently transfected with NISCH and an expression vector (NISCH (Myc-DDK-tagged)-Human nischairn, Origene, Rockville, MD, USA) using the TurboFect transfection reagent (Thermo Fisher Scientific, Waltham, MA, USA). After 24 h of incubation, the cells were administrated with diosmin at the determined concentrations. Successful transfection of CHO-K1 cells with the I-R gene (NISCH) was confirmed with Western blots.

### 2.9. Measurement of Intracellular Calcium Concentrations

The changes in the intracellular calcium concentration were detected using the fluorescent probe fura-2 [14]. NISCH-CHO-K1 cells were placed in a buffered physiological saline solution (PSS) as described previously [13]. Fura 2 (5 mM) was added to 1 mL of the cell suspension (1 × 10^6^ cells) and incubated for 30 min at 37 °C in the dark. The fluorescence was continuously recorded using a fluorescence spectrofluorometer (Hitachi F-2000, Tokyo, Japan). Values of [Ca^2+^]i were then determined, and the background measured in unloaded cells was subtracted from all measurements according to our previous report [13].

### 2.10. Western Blotting Analysis

The expression of NISCH in CHO cells was examined using Western blots, according to our recent report [15]. The CHO cells (1 × 10^6^ cells) were lysed in 1 mL of the ice-cold lysis buffer. Moreover, liver tissues were homogenated for assaying of PEPCK expression, as described in our previously report [7]. Briefly, the protein extraction was performed with ice-cold radio-immuno-precipitation assay (RIPA) buffer, and the BCA protein assay (Thermo Fisher Scientific Inc., Waltham, MA, USA) was used to determine the extracted protein concentrations. Then, protein samples (30 μg) were subjected to SDS-polyacrylamide gel electrophoresis (PAGE) (10% acrylamide gel) and were transferred to membranes using a Bio-Rad Trans-Blot system. The membranes were blocked with 5% non-fat milk, similar to our previous method [15], and then hybridized with a primary antibody specific to NISCH (Origene, Rockville, MD, USA) or PEPCK (Santa Cruz Biotechnology, Dallas, TX, USA) for 16 h. The membranes were then incubated with secondary antibodies for a further 3 h. Finally, the antigen–antibody complexes were detected using an ECL kit (Amersham Biosciences, Buckinghamshire, UK). Then, β-actin (Merck Millipore, Darmstadt, Germany) was used as an internal control. After the presence of the marker was verified for specificity, the immunoblots for NISCH (37 kDa), PEPCK (62 kDa) and β-actin (43 kDa) were quantified using a laser densitometer.

### 2.11. Statistical Analysis

The plasma glucose-lowering activity of diosmin is displayed as the percentage of the decrease in value from the initial glucose level, as described in our previous report [16]. The data are expressed as the mean ± standard error of the mean (SEM). Statistical analyses among multiple groups were analyzed via one-way ANOVA. Multiple comparisons were conducted via post hoc Newman–Keuls tests. The datasets of two sample groups were analyzed with independent Student’s *t*-tests. The statistical analysis software used was SPSS 21. A *p*-value of 0.05 or less was considered significant.

## 3. Results

### 3.1. Effect of Diosmin on BER Secretion from Adrenal Glands

**Figure 1:**
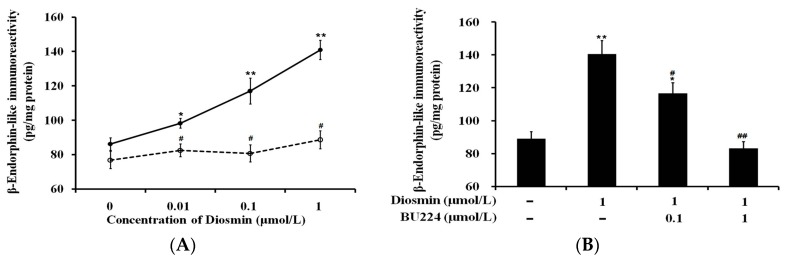
Effect of diosmin on β-endorphin secretion in isolated adrenal glands. (**A**) Dose-dependent increases in β-endorphin secretion induced by diosmin at indicated concentrations from adrenal glands in normal medium (solid line) is markedly different with that of adrenal glands incubated in calcium-free medium (broken line); (**B**) Basal β-endorphin secretion (1st column) was increased by diosmin (160 mg/kg), shown in the 2nd column, and this action was dose-dependently blocked by BU224 (columns 3 and 4) in isolated adrenal glands. The values are presented as mean ± SEM (*n* = 8). (**A**) * *p* < 0.05 and ** *p* < 0.01 compared with basal level without treatment. ^#^ *p* < 0.05 compared with the value from samples incubated at the same concentration in normal medium; (**B**) * *p* < 0.05 and ** *p* < 0.01 compared with the basal level in the 1st column. ^#^ *p* < 0.05 and ^##^ *p* < 0.01 compared with the value showing treatment with diosmin (2nd column) only.

In isolated adrenal glands, diosmin induced a marked increase of BER secretion in a dose-dependent manner (Figure 1A). However, the effectiveness of diosmin was significantly reduced in calcium-free medium (Figure 1A), indicating that the BER secretion from adrenal glands induced by diosmin is calcium dependent. 

Pretreatment with BU224, an established antagonist of I-2 receptors, inhibited the effects of diosmin in a dose-dependent manner (Figure 1B). Additionally, the inhibitory effect of BU224 disappeared after washout, and no irreversible attenuation was observed in the adrenal glands.

### 3.2. Effect of Diosmin on Calcium Concentration in NISCH-CHO-K1 Cells

**Figure 2:**
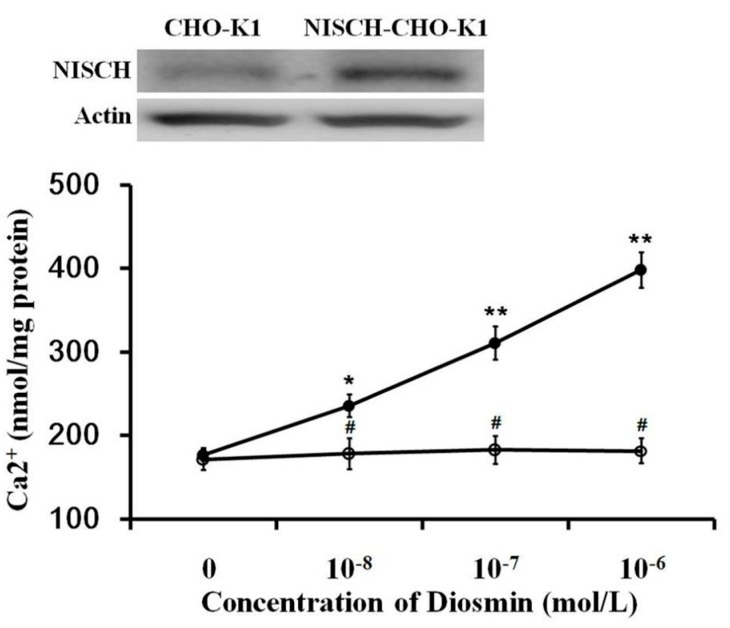
Direct effect of diosmin on imidazoline receptors (I-R) in Chinese hamster ovary (CHO-K1) cells. Successful transfection of CHO-K1 cells with the I-R gene (NISCH) is confirmed with Western blots in the upper figure. There is a dose-dependent elevation in calcium content by diosmin in NISCH-transfected CHO-K1 cells (NISCH-CHO-K1 Cells) compared with cells transfected with empty vector (CHO-K1 Cells). The values are presented as mean ± SEM (*n* = 8). * *p* < 0.05 and ** *p* < 0.01 vs. the vehicle-treated group (0 mol/L). ^#^ *p* < 0.05. vs. the values of NISCH-CHO-K1 Cells.

Following our previous method [13], the exogenous NISCH gene was transfected into CHO-K1 cells. Success of the transfection was confirmed using Western blots, as shown in the upper section of Figure 2. The expression I-R in NISCH-CHO-K1 cells is functional, as described previously [13]. 

Next, the possible effect of diosmin on I-R was evaluated. After incubation with diosmin, the calcium concentration was markedly raised in NISCH-CHO-K1 cells in a dose-dependent manner (Figure 2). However, the increase in intracellular calcium concentration by diosmin was not observed in the untransfected CHO-K1 cells (Figure 2). Therefore, a direct effect of diosmin on I-R has been identified.

### 3.3. Effects of an I-2R Blockade on Diosmin-Induced Changes in Plasma Glucsoe and BER Levels in STZ-Diabetic Rats 

**Figure 3:**
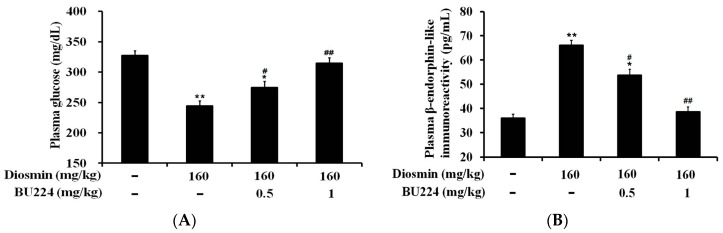
Effects of diosmin on plasma glucose and β-endorphin levels were inhibited by the blockade of I-2 receptors using an antagonist. (**A**) Hyperglycemia (1st column) was reduced by diosmin, shown in the 2nd column, and this effect was dose-dependently inhibited by the blockade of I-2R using the antagonist BU224 (columns 3 and 4) in diabetic rats; (**B**) Plasma β-endorphin (1st column) was increased by diosmin (2nd column), and this effect was dose-dependently inhibited by the blockade of I-2R using the antagonist BU224 (columns 3 and 4) in diabetic rats. The values are presented as mean ± SEM (*n* = 8). * *p* < 0.05 and ** *p* < 0.01 compared with the indicated basal level of vehicle-treated diabetic rats in the 1st column. ^#^ *p* < 0.05 and ^##^ *p* < 0.01 compared with the value resulting from the treatment with diosmin (2nd column) only.

As in our previous report [7], diosmin produced a glucose-lowering effect in STZ-diabetic rats. Then, a 30 min pretreatment with BU224, an I-2R antagonist, was performed for comparison with the vehicle-treated control. As shown in Figure 3A, hyperglycemia attenuated by diosmin was reversed by BU224 in a dose-dependent fashion. Otherwise, the plasma insulin level, which significantly decreased in STZ-diabetic rats, was not influenced by the acute treatment with diosmin [7]. Moreover, plasma BER, which increased with diosmin treatment, was also attenuated by BU224 in same manner (Figure 3B). Therefore, it is reasonable to speculate that the effects of diosmin are associated with I-2R in STZ-diabetic rats.

### 3.4. Effect of Diosmin on Plasma Glucose and Insulin Levels, Body Weight, and Food Intake in STZ-Diabetic Rats

After a 7-day continuous treatment of diosmin in STZ-diabetic rats, the hyperglycemia was significantly improved as compared to vehicle-treated group (*p* < 0.05). However, the plasma insulin level, body weight, and food intake were not changed by diosmin, compared with the vehicle-treated group Table 1.

### 3.5. Effects of I-2R Blockade on Diosmin-Induced Changes in Hepatic Glycogen Level in STZ-Diabetic Rats 

**Figure 4:**
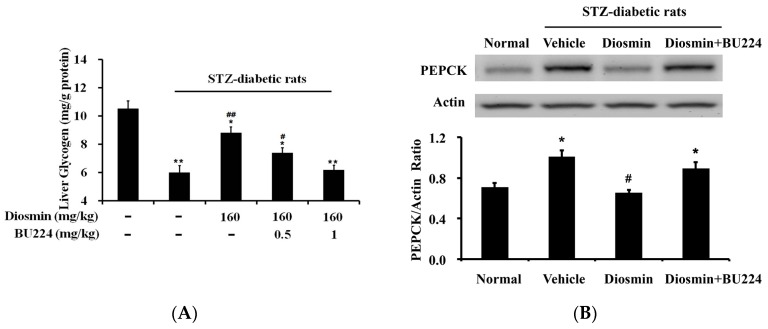
Effects of diosmin on the hepatic glycogen level was reduced by the blockade of I-2 receptors using an antagonist. (**A**) Hepatic glycogen (1st column) was reduced in diabetic rats (2nd column), and was increased by diosmin, shown in the 3rd column. This effect was dose-dependently inhibited by the blockade of I-2R using the antagonist BU224 (columns 4 and 5). The values are presented as mean ± SEM (*n* = 8). * *p* < 0.05 and ** *p* < 0.01 compared with the basal value from vehicle-treated normal rats shown in the 1st column. ^#^ *p* < 0.05 and ^##^ *p* < 0.01 compared with the value resulting from the treatment with diosmin (3rd column) only; (**B**) The expression of PEPCK in livers after same treatment with diosmin while the representative expression was showed in the upper. * *p* < 0.05 compared with the basal value from vehicle-treated normal rats shown in the 1st column. ^#^ *p* < 0.05 compared with the value in the vehicle-treated diabetic rats (2nd column).

Repeated treatment of STZ-diabetic rats with diosmin (160 mg/kg) once daily for one week resulted in a marked reduction of hyperglycemia as described in our previous report [7]. In the present study, hepatic glycogen level was markedly reduced in STZ-diabetic rats (Figure 4A). Repeated treatment with diosmin significantly increased hepatic glycogen levels. Moreover, pretreatment of diabetic rats with BU224 inhibited this effect of diosmin (Figure 4A). Otherwise, PEPCK is a key enzyme in the regulation of glucose synthesis in the liver [17]. Consistent to a previous report [18], hepatic PEPCK expression was increased in diabetic animals. Similar to our previous report [7], one week treatment with diosmin (160 mg/kg/day) attenuated the increased expression of PEPCK in the liver of diabetic rats. Additionally, the diosmin-inhibited PEPCK expression was also reversed by pretreatment with BU224 at the dose (1 mg/kg) sufficient to block I-2R (Figure 4B). Moreover, lipid levels in the livers (mg/g tissue) were also markedly increased in diabetic rats; the total cholesterol level was significantly (*p* < 0.05) raised form 7.02 ± 0.79 mg/g (*n* = 8) to 14.28 ± 1.24 mg/g (*n* = 8). Diosmin treatment (160 mg/kg/day for one week) attenuated it to 7.15 ± 0.85 mg/g (*n* = 8) that was also reversed by pretreatment with BU224 (1 mg/kg) to 14.14 ± 1.19 mg/g (*n* = 8). Similarly, hepatic triglyceride level was modified by diosmin in the same manner; the increased triglyceride (6.63 ± 0.75 mg/g) in diabetic rats (*n* = 8), compared to normal level (3.37 ± 0.69 mg/g; *n* = 8), was markedly (*p* < 0.05) reduced to 3.69 ± 0.69 mg/g (*n* = 8) by diosmin treatment. However, BU224 pretreatment also reversed the effects of diosmin to 6.11 ± 0.82 mg/g (*n* = 8) in diabetic rats. 

### 3.6. Effects of an I-2R Blockade on Diosmin-Induced Changes in Plasma Lipid Level in STZ-Diabetic Rats 

**Figure 5:**
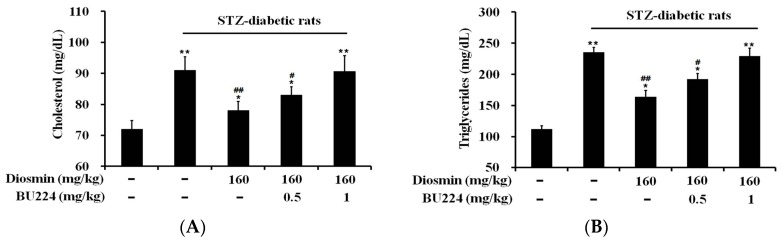
Effects of diosmin on the plasma lipid level was reduced by the blockade of I-2 receptors using an antagonist. Plasma cholesterol (**A**) and triglyceride (**B**) levels shown in the 1st column, which was elevated in diabetic rats (2nd column), was inhibited by diosmin, as shown in the 3rd column, and this effect was dose-dependently reversed by the blockade of I-2R using the antagonist BU224 (column 4th and 5th). The values are presented as mean ± SEM (*n* = 8). * *p* < 0.05 and ** *p* < 0.01 compared with the indicated basal value in vehicle-treated normal rats as shown in the 1st column. ^#^ *p* < 0.05 and ^##^ *p* < 0.01 compared with the value resulting from the treatment with diosmin (3rd column) only.

We also isolated blood samples from STZ-diabetic rats that received the repeated treatment with diosmin (160 mg/kg) once daily for one week to assay the plasma lipids. The plasma cholesterol level was markedly elevated in STZ-diabetic rats (Figure 5A). Similar to the changes in plasma glucose, the total cholesterol level was significantly attenuated by diosmin, and this effect was reversed by BU224 in a dose-dependent manner (Figure 5A). Additionally, the same changes in plasma triglyceride level were observed in STZ-diabetic rats (Figure 5B).

## 4. Discussion

In the present study, we identified the mechanism of I-2R in the effects of diosmin via intraperitoneal injection in STZ-diabetic rats, an established type-1 diabetes animal model with insulin-deficiency [19]. Additionally, we demonstrated the direct effect of diosmin on I-R using the cells that received the transfection to express I-R. 

It has been indicated that diosmin induces the reduction of hyperglycemia in STZ-diabetic rats through β-endorphin secreted from the adrenal gland [7]. Thus, following our previous method [20], we isolated the adrenal glands to investigate the potential mechanisms of diosmin. A dose-dependent increase in β-endorphin secretion from the isolated adrenal gland was observed in diosmin-treated samples, consistent with our previous report [7]. Additionally, β-endorphin secretion by diosmin was induced in a calcium-dependent manner, which is fully the same as described in a previous review [21]. Moreover, the regulatory role of I-2R in opioid secretion has been implicated in the adrenal gland [9]. Then, we applied the well-known antagonist of I-2R, BU224 [22], to confirm it. Effects of diosmin were markedly inhibited by the pretreatment with BU224 in a dose-dependent manner. Pharmacologically, it can be speculated that the mediation of I-2R is one of the effects of diosmin. Also, the I-R gene was transfected into CHO cells to prepare a functional receptor. The direct effect of diosmin on calcium influx was identified using the CHO cells expressing I-R. Therefore, for the first time, we demonstrated that diosmin effectively activates I-R.

Imidazoline receptors (I-R), also known as imidazoline/guanidinium receptive sites, are known as G-protein-coupled receptors (GPCRs) associated with glucose metabolism in peripheral tissues [23]. Three subtypes of I-R have been characterized: I-1 receptor (I-1R) activation regulates blood pressure [24], I-3 receptor (I-3R) mediates insulin release [25], and I-2 receptor (I-2R) activation increases glucose uptake into muscle cells [26,27]. However, the receptor protein of I-R is still not cloned, and most researchers used Nischarin (NISCH), a mouse homologue of human imidazoline receptor antisera-selective (IRAS) protein, instead, as described previously [13]. In the present study, we identified diosmin as an agonist of I-2R in diabetic rats. An antagonist of I-2R blocked the reduction of hyperglycemia and increase in plasma opioids induced by diosmin in a dose-dependent manner. Furthermore, diosmin alleviated the decrease in hepatic glycogen and attenuated the increased hepatic expressions of PEPCK, as described in our previous report [7], in diabetic rats that were also reversed by the blockade of I-2R. Similar changes were observed in hepatic lipids, and diosmin-induced inhibitions were reversed by antagonist of I-2R in STZ-diabetic rats. Therefore, it is reasonable to conclude that diosmin induces opioid secretion from adrenal gland via activation of I-2R to inhibit hepatic gluconeogenesis, resulting in the recovery of hepatic glycogen, which was decreased in diabetic rats. Similarly, plasma lipids, which were increased in diabetic rats, were also reduced by diosmin, and this effect was also pharmacologically inhibited by the I-2R blockade. Taken together, diosmin improves metabolic disorders mainly through an activation of I-2R in diabetic rats, particularly in the insulin-deficient state [28].

Similar to our results, oral treatment with diosmin (100 mg/kg/day) for 45 days showed a marked amelioration of STZ-induced diabetes in rats [5]. In the clinic [29], diosmin is used to treat hemorrhoids under the generic name daflon (Lab. Servier, Orléans, France). After oral administration of 10 mg/kg diosmin to healthy volunteers for 1 h, the concentration of diosmin in human plasma was 417 ± 94.1 ng/mL, which was slowly decreased after 2 h [30]. In addition to anti-inflammatory effects [31], diosmin reinforces venous tone by prolonging the activity of parietal norepinephrine [32]. Basically, diosmin belongs to a safety profile in animal studies [33] and is included in the European Pharmacopoeia. Diosmin has been clinically introduced for the treatment of venous insufficiency [34]. Diosmin has also been found to improve venous tone [35] and protect capillary bed microcirculation [36]. Additionally, oral administration of diosmin for 7 days (50 or 100 mg/kg/day) improved the cardiac functions in an ischemia–reperfusion-related cardiac dysfunction model [37]. Cardiovascular dysfunction is a common complication in diabetes [38], and diosmin seems not only to be useful for reducing hyperglycemia, but also to be advantageous for the improvement of cardiovascular disorders. Although diosmin alleviates diabetes in the present study, the effective dose of diosmin in diabetic patients is still unclear. Therefore, the useful dose of diosmin in diabetes shall be evaluated in future work. Moreover, whether the clinical merit of diosmin is associated with I-R should be investigated in the future.

## 5. Conclusions

The data obtained suggest that diosmin may enhance the secretion of endogenous β-endorphin from adrenal glands via activation of I-2R in STZ-diabetic rats. The reduction of hyperglycemia by diosmin is produced mainly through the released β-endorphin, which can activate opioid receptors to attenuate gluconeogenesis in the liver. Therefore, the low hepatic glycogen was raised by diosmin via I-2R activation in STZ-diabetic rats. Additionally, plasma lipids were also alleviated by diosmin through I-2R activation in same manner. Taken together, diosmin is suitable for development as an adjuvant to reduce hyperglycemia and plasma lipids in diabetes and/or other disorders. 

## Figures and Tables

**Table 1:** Effects of diosmin on the changes in blood glucose and insulin levels, body weight, and food intake in rats.

Group	Control (160/mg/kg/Day)	STZ	STZ + Diosmin
Blood glucose (mg/dL)	110.24 ± 7.23	339.83 ± 16.72 **	273.86 ± 16.07 **^#^
Plasma Insulin (µU/mL)	11.54 ± 2.22	2.32 ± 0.63 **	2.27 ± 0.78 **
Body weight (g)	358.24 ± 17.01	319.14 ± 12.25 **	316.78 ± 13.77 **

Values are means ± SD (*n* = 8). Diosmin (160 mg/kg) was treated once daily for seven days. * *p* < 0.05 and ** *p* < 0.01 compared with the values obtained from the normal rats (control). ^#^ *p* < 0.05 compared with the values obtained from the vehicle-treated STZ-induced diabetic rats (STZ).
